# Quantitative sensory testing in atypical odontalgia patients after local anesthesia

**DOI:** 10.1186/1129-2377-14-S1-P159

**Published:** 2013-02-21

**Authors:** AL Porporatti, PCR Conti

**Affiliations:** 1 Bauru School of Dentistry - Brazil, Brazil

## Introduction

Atypical Odontalgia (AO) is a term applied to a continuous pain in the teeth or in the tooth socket after extraction in the absence of any identifiable dental cause, under the International Classification of Headache Disorders (ICHD) and somatosensory abnormalities are common features in these patients, where Quantitative Sensory Testing (QST) are helpful tools to evaluate these cases.

## Objectives

The aim of the pilot study was to determine the effect of topical application of anesthetic cream in quantitative sensory testing (QST) findings of AO patients.

Methods Ten (7 women and 3 men) consecutive AO patients (60±14,97 years) with mean pain duration of 2.7 years (range 1-10 years were recruited from Bauru School of Dentistry, University of São Paulo (Brazil). QST was performed in all patients at baseline and 3 minutes after topical application of anesthetic cream (Benzocaine 2% - Benzotop 200mg/g, DFL). QST included tests of mechanical detection threshold (MDT) and mechanical pain threshold (MPT) with von Frey monofilaments, dynamic mechanical allodynia with cotton swab (DMA1) and with toothbrush (DMA2), heat pain thermal detection (HPD), cold pain thermal detection (CPD), temporal summation (WUR) and controlled pain modulation (CPM). The present pain intensity was also recorded with visual analogue scale (VAS). Results were analyzed with non-parametric Wilcoxon test with significance level of 5%.

## Results

QST mean values showed no differences after topical application of anesthetic cream, except for DMA2 (p=0.02) and WUR (p=0.02). Moreover mean pain in visual analogue scale relieved from 6,03 to 2,12 (64,84%; p=0.02).

## Conclusion

In this pilot study significant changes in intraoral somatosensory function were observed in AO after topical application of anesthetic cream for dynamic mechanical allodynia, associated to a reduction of pain intensity. These results may reflect peripheral and central sensitization of trigeminal pathways.

## Conflict of interest

There were no conficts of interest in the performance of this study.
